# Maintaining a young self-concept: Feeling young or shifting age thresholds?

**DOI:** 10.1007/s10433-025-00851-3

**Published:** 2025-04-03

**Authors:** Fiona S. Rupprecht, M. Clara P. de Paula Couto, Klaus Rothermund, Jana Nikitin

**Affiliations:** 1 Department of Developmental and Educational Psychology, Faculty of Psychology, University of Vienna, Vienna, Austria; 2 Department of Psychology, Friedrich Schiller University of Jena, Jena, Germany

**Keywords:** Age, Subjective age, Perceived onset of old age, Age identity, Assimilation, Accommodation

## Abstract

In the face of prevailing negative views on old age, aging individuals try to maintain the self-concept of a *young* person. They may do so by feeling younger than they are or by shifting the threshold of old age. According to the dual-process theory of developmental regulation, the former represents an assimilative coping process, whereas the latter represents an accommodative coping process. In the present work, we investigated the usage of those two processes across various life domains (e.g., family, work, and leisure). We hypothesized that individuals try to maintain a *young* self-concept particularly in those domains they view as important for themselves. We furthermore expected older adults to rely more strongly on shifting the threshold of old age in personally important domains (= accommodative response) and younger adults to rely more strongly on feeling young in personally important domains (= assimilative response). We investigated these hypotheses in a cross-sectional sample of 768 individuals aged 30 to 80 years. Analyses were conducted in a multilevel framework with the life domains nested within individuals. Our hypotheses were confirmed. Participants protected a *young* self-concept selectively in domains that were important to them. Younger adults felt particularly young in self-relevant domains, whereas older adults selectively chose higher old age thresholds in self-relevant domains. Both processes may allow individuals to protect their self-concept and to dissociate themselves from the stigmatized identity of an *old* person. Results also shed light on the idealization of being *young* in our society.

**Supplementary Information:**

The online version contains supplementary material available at 10.1007/s10433-025-00851-3.

## Introduction

When asked for the age they feel like and identify with, individuals start to state a younger age than their actual one in their mid-twenties (Rubin and Berntsen 2006; Pinquart and Wahl 2021). It is assumed that this is both reflective of a healthy and positive aging process (Stephan et al. 2015; Wurm et al. 2017), as well as adaptive because it allows individuals to dissociate themselves from the negative connotations and stereotypes associated with growing older (Weiss and Lang 2012; Weiss and Freund 2012). By feeling young irrespective of chronological age, individuals protect their self-concept of a *young* person. An alternative way to protect a young self-concept is redefining what it means to be old, that is, pushing the threshold of old age to increasingly higher ages (Chopik et al. 2018; Barrett and Toothman 2014; Wettstein et al. 2024). In modern societies, which tend to idealize youth, express serious ageism and devalue old age (North and Fiske 2015), feeling young and increasing the threshold of old age may both be prevalent responses to not identify as old and protect one’s self-concept as a young person. In the scope of this study, we investigate whether these two responses (i.e., feeling young and increasing the subjective threshold of old age) are particularly common in life domains that are personally important to the individual and thus, crucial for the respective self-concept. We also address whether chronological age is a decisive factor in which of the two responses individuals engage in. By studying how individuals maintain a young self-concept, we hope to better understand the ways in which individuals navigate an ageist world.

### Domain-specific aspects and the role of importance

Individuals differentiate between life domains (e.g., work, leisure, health, or family) when it comes to how old they feel (Kornadt et al. 2018) and to the age thresholds at which they consider a person old (Kornadt and Rothermund 2011). They tend to feel young in one domain, but old in another and perceive younger age thresholds for the domain of work than for example the domain of family (Kornadt and Rothermund 2011). Individuals may hereby feel younger or have relatively higher age thresholds in domains that are of high importance to themselves and thus, contribute more strongly to their self-concept as a young (or old) person. This perspective is based on the importance effect, which suggests that domains that individuals consider important contribute more strongly to their self-concept and self-esteem than domains they consider unimportant (Hardy and Moriarty 2006; Marsh 1986).

When a *young* self-concept is threatened in such important and self-relevant domains, individuals can respond in either assimilative or accommodative ways (Brandtstädter and Rothermund 1994, 2002). Assimilative processes comprise the selective investment of resources to modify the status quo according to one’s personal goals and values. In self-relevant domains, individuals may thus maintain or build up *young* activities and roles, *young* relationships, or *young* appearances, which allow them to (continue to) feel young in consequence. Accommodation, on the other hand, comprises processes of reappraisal and adjustment of personal goals and aspirations to what is feasible in order to maintain a positive self-concept despite restricted resources and possibilities. Against the inadvertent increase in one’s own chronological age, individuals can redefine what it means to be *old* in personally important domains and adapt age thresholds accordingly. In domains that are less important and self-relevant, the dual-process model of assimilation and accommodation predicts that individuals should not aim at maintaining a *young* self-concept, but figuratively grow old, and adopt the self-image of an *old* person earlier (cf. Brandtstädter and Rothermund 2002). Our main hypothesis is thus that when life domains are personally important, individuals defend a *young* self-concept in these domains by drawing on either assimilative or accommodative processes—investing in feeling young or increasing the threshold of old age.

### The moderating function of chronological age

In order to protect their self-concept of a *young* person, individuals may respond in two different ways (i.e., trying to maintain youthful attributes in order to feel younger or adapting the age threshold). Chronological age should be a decisive factor for which of the two responses an individual is more likely to use, with accommodative processes gaining importance and replacing assimilative processes with increasing age (Brandtstädter and Rothermund 2002; Rothermund and Brandtstädter 2003). Age-related changes such as retirement, personal illness, and the death of loved ones may restrain assimilative efforts (Rothermund and Brandtstädter 2003), make it more difficult, effortful and less efficient to actively invest in a young self-concept, and lead individuals to focus on the reappraisal of personal and societal standards of being considered old instead. As individuals grow older, age thresholds also become increasingly closer and imminent, which may further foster an accommodative response. Older adults may thus focus more strongly on adapting age thresholds than younger adults, for whom these thresholds are still distant and mostly irrelevant.

Empirical research supports this claim in a general sense: Individuals heighten their age thresholds as they grow older and do so particularly at advanced ages (Barrett and Toothman 2014; Ennis et al. 2024; Kornadt and Rothermund 2011; Wettstein et al. 2024). In contrast, older adults appear to be more set and stable in their felt ages, whereas younger adults’ felt ages are more malleable and probably more easily adapted (Bellingtier et al. 2021). Adding a domain-specific perspective, we hypothesize selective efforts, that is, efforts driven by personal goals, values, and the self-assigned importance of a domain, in the response mode that is natural to the respective phase of life. We expect the assimilative response of feeling younger in personally important domains to be more prominent among younger adults and the accommodative response of adapting age thresholds in personally important domains to be more prominent among older adults.

### The present study

To test our hypotheses, we conducted a cross sectional analysis in an adult lifespan sample drawn from the Aging as Future project (Lang et al. 2024). The study applies a domain-specific and idiographic perspective as proposed by researchers studying importance theory (Hardy and Moriarty 2006): When specific domains are more important to the individual than other domains, the responses for protecting the self-concept of a *young* person should be more prominent. We thus expected to find lower felt ages and higher age thresholds in the domains that are of relatively higher importance to the individual. Additionally, we investigated whether chronological age would moderate the relationship between importance and felt age/age thresholds in that higher domain-specific importance would be associated with particularly high age thresholds among older adults but particularly low felt ages among younger adults.

## Method

### Transparency, openness, and data availability

We used the first wave paper–pencil data from the Aging as Future project (Lang et al. 2024). The data is currently in the process of being published. Our statistical code will be made available upon request. The study design, hypotheses, and analytic plan were not preregistered. The following section reports the sample size, any data exclusions, all data preparations, and all measures that were used for our analyses.

### Sample and procedure

Participants were recruited via local registry offices in two middle-sized German cities (Jena for East Germany and Erlangen for West Germany). Data were stratified by birth cohort and gender and individuals were invited using postcards, which they had to send back in order to receive the paper–pencil questionnaires. The response rate to the postcards amounted to 21% and out of the initially contacted individuals 19% filled in the questionnaire. Participation was rewarded with a charity lottery ticket worth 8€ (for more information on study recruitment and procedure see Kornadt and Rothermund 2011). The sample consisted of 768 individuals who participated in the study in 2009. Participants were aged between 30 and 80 years old (*M* = 55.3, *SD* = 14.9). Half of the sample (*n* = 380, 49%) were women, 31% had vocational secondary education, 18% had vocational tertiary education, and 46% had university-level tertiary education. 10% were single, 74% were married or in a partnership, 10% were separated or divorced, and 6% were widowed. 52% of the sample were currently employed, 39% were retired and further 9% were currently unemployed. Research procedures were approved by the Institutional Review Board at Friedrich Schiller University Jena (FSV 18/36).

### Measures

Apart from chronological age, all variables were measured specifically within the following eight domains (Kornadt and Rothermund 2011): Family and partnership; friends and acquaintances; religion and spirituality; leisure activities and social or civic commitment; personality and way of living; financial situation and dealing with money-related issues; work and employment; physical and mental fitness, health and appearance. The domains were selected based on the results of a literature search and a pilot study, in order to identify domains that are relevant for older individuals and in which age-related changes (gains and/or losses) are expected to occur. In the pilot study, a convenience sample of older adults (*n* = 14) were interviewed in an open format and reported on their beliefs and attitudes on actual and expected changes and shifts in importance in various life domains that, in their opinion, were due to age and the aging process. The answers were thematically grouped, and domains for the final study were selected that were linked most often to age and age-related changes. Interview statements were also used to phrase specific items for the assessment of beliefs, and importance ratings within each domain (for details, see Kornadt & Rothermund 2011).

*Importance.* Importance was assessed with three to four items per domain. These items were derived from the scales measuring domain-specific views on aging developed by Kornadt and Rothermund (2011). Each item statement in the views on aging scales was hence adapted and presented in the importance measure. Items started with the phrase “How important is it to you…” and ended with domain-specific statements. In the domain of *personality and way of living* these statements were for example “…to deal with problems calmly and considerately”, “…to find the correct solution for important life questions”, and “…to be open-minded and tolerant”. Items were answered on a scale ranging from “not at all important” (0) to “very important” (4). Within-domain reliabilities of the importance scores were acceptable for most domains (Cronbach’s alpha of 0.67 for *family and partnership*, 0.72 for *friends and acquaintances*, 0.78 for *religion and spirituality*, 0.81 for l*eisure activities and social or civic commitment*, 0.71 for *personality and way of living*, and 0.74 for *health and appearance*) except for *financial situation and dealing with money-related issues* (*α* = 0.47) as well as *work and employment* (*α* = 0.41), where statements appeared more heterogeneous. Despite low reliabilities in these two domains, we decided to proceed with the respective three items to not lose the breadth of the importance measure.

*Felt Age.* Felt age was assessed with the question “What age group would you compare yourself with in the domain of X? In the domain of X, I feel as though I am ____ years old”. In a first step, we excluded answers that would not be meaningful in the context of our analyses, that is ages above the human life expectancy (i.e., > 125 years, *n* = 1) and ages below the legal entry of adulthood (i.e., < 18 years, *n* = 12), which may indicate feeling like a child rather than feeling young(er). We then computed a proportional discrepancy score [(felt age – age) / age] that represents by how many percent individuals feel younger or older than they currently are. This approach is recommended particularly for samples with wide age ranges such as ours as it relativizes the discrepancy on the years an individual has already lived (e.g., feeling 20 years younger may mean something else for a 90-year old compared to a 40-year old; Kotter-Grühn et al. 2015). In order to ease the interpretation of regression coefficients, we again multiplied the felt age proportional discrepancy by 100, so that a value of 15 would indicate that an individual feels 15% older than they actually are.

*Age Threshold.* Age thresholds were assessed with the question “From what age on would you consider a person as old in the domain X?” and individuals gave their answer in years.

*Age.* Chronological age was assessed as years since birth.

*Covariates.* Sex, health, education, employment status, and relationship status may be related to how old individuals feel and how they define old age (Bellingtier et al. 2021; Wettstein et al. 2024) and were thus added as covariates. Sex was coded as *female* (0) and *male* (1), self-rated health was assessed on a scale ranging from *not good at all* (0) to *very good* (4), education was coded as *no university-level education* (0) and *university-level education* (1), employment status was coded as *unemployed (including retired)* (0) and *employed* (1), and relationship status was coded as *not living in a partnership* (0) and *living in a partnership* (1).

### Data analyses

Felt age and age threshold served as outcome variables and importance and chronological age as predictors. Except for chronological age and the covariates, all variables were domain-specific, meaning that each individual had scores in several domains (e.g., importance for each of the eight domains). We thus applied a multilevel structure with the individual on Level 2 and the domain-specific constructs nested within the individual on Level 1. This approach is in accordance with both the idiographic approach suggested for the importance hypothesis (Hardy and Moriarty 2006) and the domain-specific modeling we have applied in past publications using a different data set (Kim-Knauss et al. 2022; Lang and Rupprecht 2021).

We analyzed our hypotheses in four steps: Model 1 estimates an intercept and includes differences between the eight domains. We chose family and partnership as the reference category because felt ages were lowest and age thresholds were highest here (see Table 1). The intercept in the models thus refers to the value in the family domain and the seven domain coefficients refer to domain-specific deviations in felt age and age threshold. Model 2 adds importance and chronological age as predictors. The two linear predictors were mean-centered before being entered into the models. Model 3 adds a random slope in importance and the interaction between importance and chronological age. Model 4, which can be found in the supplement, adds the covariates sex, health, education, employment status, and marital status. The model equation for Model 3 with felt age as the outcome variable is the following:

$${\text{Felt Age}}_{{{\text{id}}}} = {\text{g}}_{00} + {\text{g}}_{{{1}0}} {\text{friendships}}_{{{\text{id}}}} + {\text{g}}_{{{2}0}} {\text{religiosity}}_{{{\text{id}}}} + {\text{g}}_{{{3}0}} {\text{leisure}}_{{{\text{id}}}} + {\text{g}}_{{{4}0}} {\text{personality}}_{{{\text{id}}}} + {\text{g}}_{{{5}0}} {\text{finances}}_{{{\text{id}}}} + {\text{g}}_{{{6}0}} {\text{work}}_{{{\text{id}}}} + {\text{g}}_{{{7}0}} {\text{health}}_{{{\text{id}}}} + {\text{g}}_{{{8}0}} {\text{importance}}_{{{\text{id}}}} + {\text{g}}_{{0{1}}} {\text{age}}_{{\text{i}}} + {\text{g}}_{{{81}}} {\text{age}}_{{\text{i}}} *{\text{importance}}_{{{\text{id}}}} + {\text{U}}_{{0{\text{i}}}} + {\text{U}}_{{{\text{8i}}}} + {\text{ R}}_{{{\text{it}}}}$$

**Table 1:** Domain-specific means and standard deviations

Variable	Felt age*M* (*SD*)	Age threshold *M* (*SD*)	Importance*M* (*SD*)
Family	**− **12.38 (16.99)	69.90 (7.71)	3.46 (0.62)
Friendships	**− **9.71 (19.76)	67.74 (9.28)	2.52 (0.78)
Religiosity	**− **7.27 (24.97)	66.65 (10.18)	1.70 (1.06)
Leisure	**− **7.59 (20.16)	65.78 (9.51)	2.56 (0.73)
Personality	**− **6.89 (20.01)	65.64 (9.65)	3.29 (0.56)
Finances	**− **3.62 (22.39)	65.05 (8.14)	2.32 (0.69)
Work	**− **8.12 (15.14)	60.48 (7.39)	3.00 (0.58)
Health	**− **9.00 (17.71)	66.37 (9.25)	3.53 (0.49)
Global	**− **8.07 (19.95)	65.95 (9.28)	2.80 (0.93)

Felt age is giving in percent an individual felt younger than their chronological age. Age threshold is given in absolute years. Importance was reported on a scale ranging from 0 to 4. “Global” refers to the across domain averages

The felt age of individual i in the domain d equals the average intercept γ_00_ (for the domain of family) plus the domain-specific deviation γ_10_ to γ_70_ from the intercept, the intraindividual (i.e., Level 1) effect of importance γ_80_, the interindividual (i.e., Level 2) effect of age γ_01_, the cross-level interaction of age and importance γ_81_, the individual deviation from the intercept U_0i_ (i.e., random intercept), the individual deviation from the importance effect U_8i_ (i.e., random slope of importance), and the individual error term R_it_.

## Results

Table 1 reports the domain-specific descriptive statistics for felt age, age threshold, and importance. Felt age was lowest in the domain of family and highest in the domain of finances. The felt age variable had an ICC of 0.29, indicating that 29% of variance in felt age was shared across domains, whereas 71% of variance was domain-specific. Notably, within-domain standard deviations were high for felt age, which could indicate both the large age range of the sample as well as an individualized perception of the single domains. The old age threshold was lowest in the work domain and highest in the family domain. The age threshold variable had an ICC of 0.38, indicating a somewhat higher amount of shared variance across domains (38%), while 62% of variance was domain-specific. Felt age and age threshold were unrelated to each other on the individual level, *r* = − 0.08, *p* = 0.080, and negligibly related on the domain-specific level, *r* = − 0.03, *p* = 0.017. Importance was highest in the domain of health and lowest in the domain of religiosity. The importance variable had an ICC of 0.05, indicating that there was only very little shared variance (5%), but that importance was a highly domain-specific construct. In other words, nearly all individuals considered specific domains more important and other domains less important, which is further validation for a domain-specific and idiographic approach.

**Fig. 1:**
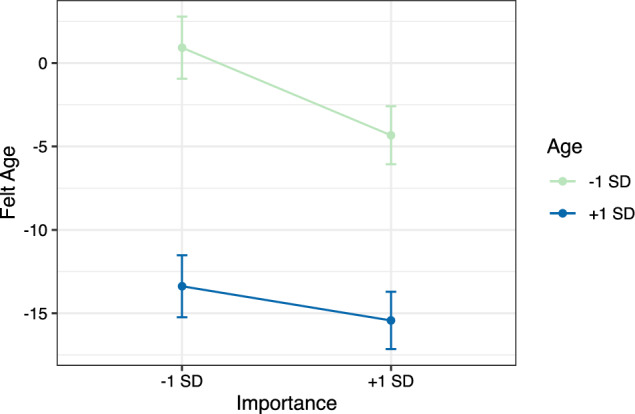
Felt Ages in Association with Age and Importance. Descriptive Caption. At younger ages, there is a significant importance effect in that felt age is younger in personally important domains. Importance hereby seems decisive whether a young adult feels younger or feels their age in a specific domain. At older ages, there is no significant effect of importance on felt age, instead older adults seem to feel much younger in general and across domains

In a next step, we built the multilevel models which can be seen in Table 2. For felt age as an outcome, Model 1a gives essentially the same information that is already contained in Table 1. For Model 2a, importance and age were entered as intraindividual and interindividual predictors, respectively. Both were negatively linked with felt age, indicating that older people felt younger on average and that participants reported younger felt ages in domains they considered important. Age and importance together explained 11.0% of the variance in felt age (9.8% when accounting for the random slope in Model 2a). Model fit improved significantly, ΔChi^2^(2) = 273.54, *p* < 0.001. In Model 3a, the cross-level interaction between importance and age was entered, explaining 2.5% of variance in the random slope and adding significantly to the overall model fit, ΔChi^2^(1) = 7.64, *p* = 0.006. The significant interaction is depicted in Fig. 1. Younger adults reported feeling younger particularly in those domains they considered important for themselves. In domains they considered relatively less important, they felt close to their chronological age. In contrast, older adults felt much younger in general, largely irrespective of whether they considered a specific domain important. Johnson–Neyman intervals indicated that the relationship between importance and felt age was significant up till an age of 71 years. In Model 4a (Supplementary Table S1), all effects remained significant and the covariates explained 1.0% of additional variance, with better health and employment being linked to younger felt ages. Our hypotheses that (a) individuals felt younger in domains they considered important and (b) that this assimilative tendency was stronger among younger adults were thus both supported.

**Table 2:** Cross sectional relations between felt age and age threshold with importance and age

	Felt age			Age threshold		
	Model 1a	Model 2a	Model 3a	Model 1b	Model 2b	Model 3b
	γ (SE)	γ (SE)	γ (SE)	γ (SE)	γ (SE)	γ (SE)
Intercept	**− 12.39** (0.73)	**− 11.11** (0.71)	**− 11.11** (0.71)	**69.90** (0.32)	**69.63** (0.34)	**69.63** (0.35)
Friendship	**2.70** (0.86)	0.69 (0.89)	0.71 (0.89)	**− 2.15** (0.35)	**− 1.78** (0.38)	**− 1.77** (0.38)
Religiosity	**5.05** (0.87)	1.71 (1.04)	1.62 (1.04)	**− 3.21** (0.35)	**− 2.46** (0.43)	**− 2.51** (0.43)
Leisure	**4.90** (0.86)	**2.92** (0.89)	**2.93** (0.89)	**− 4.10** (0.35)	**− 3.67** (0.37)	**− 3.67** (0.37)
Personality	**5.49** (0.86)	**5.11** (0.84)	**5.10** (0.84)	**− 4.21** (0.35)	**− 4.10** (0.35)	**− 4.11** (0.35)
Finances	**8.75** (0.86)	**6.57** (0.92)	**6.58** (0.92)	**− 4.82** (0.35)	**− 4.31** (0.39)	**− 4.30** (0.39)
Work	**4.29** (0.86)	**3.32** (0.85)	**3.28** (0.85)	**− 9.41** (0.35)	**− 9.22** (0.36)	**− 9.24** (0.36)
Health	**3.51** (0.87)	**3.61** (0.83)	**3.61** (0.83)	**− 3.49** (0.35)	**− 3.52** (0.35)	**− 3.52** (0.35)
Importance		**− 1.98** (0.40)	**− 1.98** (0.40)		**0.45** (0.15)	**0.45** (0.15)
Age		**− 0.42** (0.03)	**− 0.43** (0.02)		**0.09** (0.01)	**0.09** (0.01)
Importance*Age			**0.06** (0.02)			**0.02** (0.01)
σ^2^_Slope_Importance_		28.60	27.89		1.61	1.51
σ^2^_Interindividual_	117.60	73.47	73.56	33.35	31.39	31.43
σ^2^_Intraindividual_	277.30	254.19	254.16	46.42	45.09	45.08

*Notes*: Significant coefficients (*p* < 0.05) are printed bold. Age is the only interindividual (i.e., Level 2) predictor, all other predictors are intraindividual (i.e., Level 1). The interaction between importance and age is a cross-level interaction. The random slope for importance was added in Models 2a and 2b. Model fit increased significantly after allowing for the random slope both in Model 2a, ΔChi^2^(2) = 124.90, *p* < 0.001, and Model 2b, ΔChi^2^(2) = 20.69, *p* < 0.001. Family and partnership serves as the reference category

**Fig. 2:**
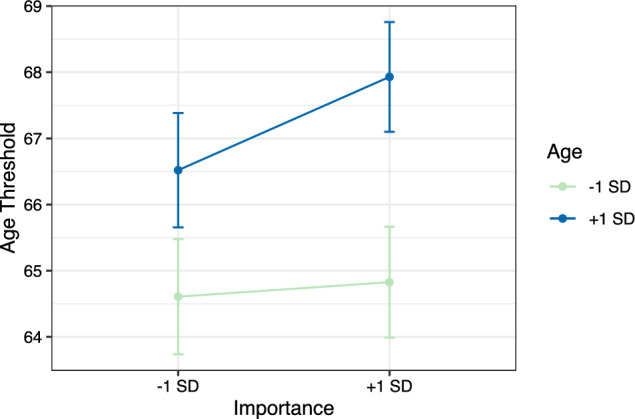
Age Thresholds in Association with Age and Importance. Descriptive Caption. At younger ages, there is no importance effect on age thresholds, instead age thresholds are non-selectively chosen around the legal retirement age of 65 years. At older ages, there is a significant effect of importance on age thresholds in that older adults set higher age thresholds in domains that are of personal importance for them

For age threshold as an outcome variable (also see Table 2), Model 1b again mirrors the descriptive statistics in Table 1. For Model 2b, age and importance were both positively associated with age thresholds, indicating that older adults reported higher age thresholds on average (i.e., old age starts later from the perspective of older compared to younger adults) and that individuals reported higher age thresholds in domains they considered important for themselves. Age and importance together explained 2.4% of variance (2.1% when accounting for the random slope in Model 2b). Model fit improved significantly, ΔChi^2^(2) = 41.02, *p* < 0.001. In Model 3b, the cross-level interaction between age and importance was entered, explaining 6.2% of variance in the random slope and adding significantly to the overall model fit, ΔChi^2^(1) = 8.22, *p* = 0.004. Figure 2 shows how younger participants chose age thresholds close to Germany’s retirement age at the time of data collection (i.e., 65 years), irrespective of the personal importance of a domain. In contrast, older adults chose higher age thresholds in domains that were important to them. Depending on the specific individual and domain in question, importance proved decisive whether older adults’ own chronological ages lay below the age threshold or above. Johnson–Neyman intervals indicated that the relationship between importance and age threshold was significant for individuals aged 50 years and older. In Model 4b (Supplementary Table S1), all effects remained significant and covariates explained 4.0% of additional variance, with male sex and unemployment being linked to lower age thresholds. Our hypotheses that (a) individuals chose higher age thresholds in domains they considered important and (b) that this accommodative tendency became stronger with advancing age were thus both supported.

## Discussion

We investigated the prevalence of assimilative (feeling younger) and accommodative (increasing age thresholds) processes to maintain a young self-concept in personally important domains. According to hypotheses, individuals indeed reported younger felt ages and higher age thresholds in domains they considered important for themselves than in domains considered unimportant. They thus seemed to protect the self-concept of a *young* person particularly in domains that were self-relevant. Younger adults did so by adopting the more assimilative mode of selectively maintaining youthful attributes and feeling young(er). Older adults, in contrast, rather expressed accommodative tendencies by selectively holding higher perceived age thresholds in self-relevant domains and thus keeping *old age* at a distance.

### Importance effect in subjective aging

The present study clearly supported prior research on the domain-specificity of subjective aging (Kornadt and Rothermund 2011), as individuals chose different felt ages and age thresholds depending on the domain. With variance shares of 71% and 62% on the level of domains, domain differentiation was high for both felt age and age threshold, though slightly lower for the latter, where interindividual covariates (i.e., sex and employment) and macro-level factors may play a larger role (Jurek 2021). It was however not only the content of the domain (e.g., family vs. work) that mattered (see the large SDs for felt age even within domains, Table 1), but also the personal importance an individual assigned to it. To the best of our knowledge, the present study is therefore the first to show an importance and selectivity effect (Hardy and Moriarty 2006; Marsh 1986; Brandtstädter and Rothermund 2002) in the area of subjective aging: Individuals reported younger felt ages and higher age thresholds in domains of personal importance. They thus *selectively* protected their young self-concept in self-relevant domains. Conversely, in domains less important to the individual, the effort to continuously feel young or to redefine old age may not be kept up, but individuals may accept their own aging process more readily. This implies that individuals may not strive for a youthful self-concept in a general manner, comprising all facets of life, but just in those that matter most to them. Next to the dual-process model of assimilation and accommodation (Brandtstädter and Rothermund 2002; Rothermund and Brandtstädter 2021), these results are in line with other major lifespan theories that place an individual’s selective and personalized engagement in specific areas of life at their heart—the selection, optimization, and compensation model (Baltes et al. 2007), models of optimal aging (Aldwin et al. 2018), as well as more recent conceptualizations of successful aging (Rowe and Kahn 2015; Sabatini et al. 2024). Targeting subjective aging idiographically rather than globally may thus be a promising avenue for future research.

### Assimilative and accommodative responses

The importance effect for felt age was strongest among the youngest participants (i.e., 30 to 40 years old). It was significant till the age of 71 years, indicating that up until early old age individuals felt youngest in the domains they considered important. Only the oldest adults in our sample (up to 80 years old) felt young across domains, irrespective of the personal importance assigned to them, which replicates the established, though seemingly non-selective main effect of age on felt age (Pinquart and Wahl 2021). Across a large part of adulthood, individuals may thus use an assimilative response and build a personalized and importance-based self-concept as a *young* person. This self-concept may gain in generality once assimilative efforts in specific domains become more difficult to uphold, for example due to age-related events such as retirement, personal illnesses, the deaths of loved ones, and one’s own impending end of life (Rothermund and Brandtstädter 2003).

In contrast, starting from the age of 50 years, the importance effect was significant for age thresholds. Participants in this age group upheld their *young* self-concept by accommodating their personal standards for the beginning of old age. They did so by generally setting higher age thresholds across domains than younger participants, which indicates the age thresholds’ increasing proximity, familiarity, and self-relevance for older adults (Wettstein et al. 2024). Older participants however adjusted age thresholds most strongly in domains they considered important for themselves, which reflects the natural shift to personalized, accommodative responses at older ages (Brandtstädter and Rothermund 2002). We did not assess the underlying processes that supported accommodative adjustments of age thresholds (e.g., raising the personal age threshold from 65 to 70 years) directly, but it seems likely that a selective focus on more negative and severe indicators of aging in the respective domains might have led to increased age thresholds. Distancing oneself from these negative images of aging allows individuals to avoid a self-categorization as old in important domains (although this not necessarily leads to feeling particularly young in these domains). Participants below the age of 50 years chose age thresholds irrespectively of personal domain importance and close to the sample-specific retirement age of 65 years. Younger participants thus seemed to use a generic anchor for the beginning of old age, which is in line with the embeddedness of age thresholds in macro-level predictors such as country-specific healthy life expectancy, retirement age, and labor market aspects (Jurek 2021; Augustynski and Jurek 2021).

Taken together, selective attempts to maintain a young self-concept in personally important domains were mostly employed via the response mode that is natural to the respective phase of life. That is, by actively investing in being *young* (i.e., assimilating) earlier in life, and by focusing on not having to categorize oneself as *old* (i.e., accommodating) later in life. Felt ages and age thresholds were unrelated both on the individual and on the domain-specific level, implying that individuals tended to maintain a young identity in either of the two ways rather than relying on both processes simultaneously. Theoretically, these results are in line with the dual-process model (Brandtstädter and Rothermund 1994, 2002), which posits that the two coping modes operate independently, with younger adults protecting their self-concept via an assimilative response (i.e., investing in youthful attributes and feeling young in personally important domains), whereas older adults doing so via an accommodative response (i.e., redefining old age and adapting age thresholds in personally important domains). Previous empirical work however showed a positive association between younger felt ages and higher age thresholds (Wettstein et al. 2024; Toothman and Barrett 2011). While these previous studies are not fully comparable to ours (i.e., specific age sample; assessment of felt age and age thresholds across domains), future research should put a dedicated focus on the flexible usage and interplay of the two responses in specific domains, at specific ages and across the entire lifespan.

### Future research and outlook

One major open question refers to the adaptive value of the processes investigated in the present study. The importance effect assumes that self-esteem is primarily derived from personally important life domains (Marsh 1986). Selectively investing one’s resources in a young self-concept may thus be an adaptive response to an ageist world—future research will however need to test this. In this vein, recent research suggests that distancing oneself from ageist views by feeling younger may only be adaptive in the short-term (Kornadt et al. 2023) and we know relatively little about the general adaptivity of keeping age thresholds at a distance (for one exception see Kuper and Marmot 2003).

Another shortcoming of the current study refers to its cross sectional character. Due to the cross sectional character it is likely that some of our age effects are actually cohort effects (Ennis et al. 2024; Wettstein et al. 2024, 2023). Although our theoretical background suggests that importance should be the predictor of selectively applied (assimilative or accommodative) processes, it could also be the other way around. If felt ages are not youthful enough or age thresholds would define the person as *old*, this could lead to a devaluing of domains as a consequence (Brandtstädter & Rothermund 1994; Rothermund & Brandtstädter 2003). Individuals may thus adapt the importance of domains to the self, which could be another form of accommodation, labeled as *identity accommodation* (Sneed and Whitbourne 2003). The devaluing of formerly important domains as well as the failure to do so could come with a range of consequences, including changes in self and self-esteem as well as emotional responses. It may be particularly interesting to see what happens from a longitudinal point of view if individuals actually cross age thresholds in personally important domains.

### Limitations

Regarding the constructs used, reliability of the importance measures was insufficient for the domains of finances and work. Reliability was likely low because importance in both domains can be derived and was assessed from a personal perspective (e.g., the importance of having plenty of money for oneself) and an interpersonal perspective (e.g., the importance of being able to support others financially).

Variance explanation was rather small, particularly in regard to age thresholds. Next to age, perceived time to death may be another likely candidate to explain whether individuals assimilate or accommodate (Rothermund and Brandtstädter 2003). While the interindividual covariates used in the supplement added slightly to the variance explanation, future research could also focus on intraindividual, domain-specific predictors and covariates, such as feelings of controllability and whether positive or negative age stereotypes are held for a specific domain.

In terms of transferability, our sample is Western European and highly educated. Both the motivation to protect a young self-concept, and the resources to do so may be different in other contexts characterized by a different culture of aging and levels of experienced ageism (de Paula Couto et al. 2023). Particularly age thresholds are rooted in time and place (Augustynski and Jurek 2021; Wettstein et al. 2024). For example, since the 2009 data collection, German retirement age has been raised to 67 years and related changes in age thresholds could be expected.

### Conclusion

In the scope of this study, we were able to show that individuals selectively hold on to young self-concepts in domains that are personally important to them. Younger adults tended to do so by feeling young(er), whereas older adults tended to do so by adapting the starting points of old age. The results illustrate how aging individuals navigate an ageist world and protect themselves in areas of life that are decisive for who they are as a person.

## Supplementary Information

Below is the link to the electronic supplementary material.

Supplementary file1 (DOCX 18 KB)

## Data Availability

We used the first wave paper–pencil data from the Aging as Future project (Lang et al. 2024). The data is currently in the process of being published.
