# Supplementary material for: Maintaining a young self-concept: Feeling young or shifting age thresholds?
Source: Eur J Ageing. 2025 Apr 3;22(1):15. doi: 10.1007/s10433-025-00851-3 (PMC11968622; doi:10.1007/s10433-025-00851-3)
Supplement: Supplementary file 1 — Supplementary file1 (DOCX 18 KB) [file 10433_2025_851_MOESM1_ESM.docx]

| **Supplementary Table S1** | | | | |
| --- | --- | --- | --- | --- |
| Main Analyses with Added Covariates. | | | | |
|  |  | Felt Age |  | Age Threshold |
|  |  | Model 4a |  | Model 4b |
|  |  | γ (SE) |  | γ (SE) |
| Intercept |  | **-11.00** (1.16) |  | **69.99** (0.63) |
|  |  |  |  |  |
| Friendship |  | 1.13 (0.92) |  | **-1.88** (0.39) |
| Religiosity |  | 2.02 (1.08) |  | **-2.52** (0.45) |
| Leisure |  | **3.25** (0.92) |  | **-3.73** (0.38) |
| Personality |  | **5.38** (0.86) |  | **-4.21** (0.36) |
| Finances |  | **6.85** (0.95) |  | **-4.39** (0.40) |
| Work |  | **3.46** (0.88) |  | **-9.22** (0.37) |
| Health |  | **3.67** (0.86) |  | **-3.62** (0.36) |
|  |  |  |  |  |
| Importance |  | **-1.74** (0.41) |  | **0.43** (0.16) |
| Age |  | **-0.52** (0.04) |  | **0.12** (0.02) |
|  |  |  |  |  |
| Importance*Age |  | **0.06** (0.02) |  | **0.02** (0.01) |
|  |  |  |  |  |
| Sex |  | -0.39 (0.77) |  | **-3.34** (0.44) |
| Health |  | **-2.17** (0.41) |  | 0.29 (0.24) |
| Education |  | 0.07 (0.77) |  | -0.06 (0.45) |
| Employment Status |  | **-2.54** (1.05) |  | **1.41** (0.61) |
| Relationship Status |  | 1.50 (0.87) |  | 0.83 (0.51) |
|  |  |  |  |  |
| σ^2^_Slope_Importance_ |  | 28.18 |  | 1.50 |
| σ^2^_Interindividual_ |  | 67.70 |  | 28.32 |
| σ^2^_Intraindividual_ |  | 256.01 |  | 44.99 |
| *Notes.* Significant coefficients (*p* < .05) are printed bold. Family and partnership serves as the reference category. Age, sex, health, education, employment status and relationship status are interindividual (i.e., Level 2) predictors, all other predictors are intraindividual (i.e., Level 1). The interaction between importance and age is a cross-level interaction. | | | | |
